# Cargo and Functional Profile of Saliva-Derived Exosomes Reveal Biomarkers Specific for Head and Neck Cancer

**DOI:** 10.3389/fmed.2022.904295

**Published:** 2022-07-11

**Authors:** Linda Hofmann, Valentin Medyany, Jasmin Ezić, Ramin Lotfi, Beate Niesler, Ralph Röth, Daphne Engelhardt, Simon Laban, Patrick J. Schuler, Thomas K. Hoffmann, Cornelia Brunner, Edwin K. Jackson, Marie-Nicole Theodoraki

**Affiliations:** ^1^Department of Otorhinolaryngology, Head and Neck Surgery, Ulm University Medical Center, Ulm, Germany; ^2^Institute for Clinical Transfusion Medicine and Immunogenetics Ulm, German Red Cross Blood Services Baden-Württemberg-Hessen, Ulm, Germany; ^3^Institute for Transfusion Medicine, University Hospital Ulm, Ulm, Germany; ^4^nCounter Core Facility, Institute of Human Genetics, University of Heidelberg, Heidelberg, Germany; ^5^Department of Pharmacology and Chemical Biology, University of Pittsburgh School of Medicine, Pittsburgh, PA, United States

**Keywords:** exosomes, head and neck squamous cell carcinoma (HNSCC), saliva, liquid biopsy, miRNA

## Abstract

**Background:**

Exosomes contribute to immunosuppression in head and neck squamous cell carcinoma (HNSCC), a tumor entity which lacks specific tumor biomarkers. Plasma-derived exosomes from HNSCC patients correlate with clinical parameters and have potential as liquid biopsy. Here, we investigate the cargo and functional profile of saliva-derived exosomes from HNSCC patients and their potential as non-invasive biomarkers for disease detection and immunomodulation.

**Methods:**

Exosomes were isolated from saliva of HNSCC patients (*n* = 21) and healthy donors (HD, *n* = 12) by differential ultracentrifugation. Surface values of immune checkpoints and tumor associated antigens on saliva-derived exosomes were analyzed by bead-based flow cytometry using CD63 capture. Upon co-incubation with saliva-derived exosomes, activity and proliferation of T cells were assessed by flow cytometry (CD69 expression, CFSE assay). Adenosine levels were measured by mass spectrometry after incubation of saliva-derived exosomes with exogenous ATP. miRNA profiling of saliva-derived exosomes was performed using the nCounter^®^ SPRINT system.

**Results:**

Saliva-derived, CD63-captured exosomes from HNSCC patients carried high amounts of CD44v3, PDL1 and CD39. Compared to plasma, saliva was rich in tumor-derived, CD44v3^+^ exosomes and poor in hematopoietic cell-derived, CD45^+^ exosomes. CD8^+^ T cell activity was attenuated by saliva-derived exosomes from HNSCC patients, while proliferation of CD4^+^ T cells was not affected. Further, saliva-derived exosomes produced high levels of immunosuppressive adenosine. 62 HD- and 31 HNSCC-exclusive miRNAs were identified. Samples were grouped in “Healthy” and “Cancer” based on their saliva-derived exosomal miRNA profile, which was further found to be involved in RAS/MAPK, NF-κB complex, Smad2/3, and IFN-α signaling.

**Conclusions:**

Saliva-derived exosomes from HNSCC patients were enriched in tumor-derived exosomes whose cargo and functional profile reflected an immunosuppressive TME. Surface values of CD44v3, PDL1 and CD39 on CD63-captured exosomes, adenosine production and the miRNA cargo of saliva-derived exosomes emerged as discriminators of disease and emphasized their potential as liquid biomarkers specific for HNSCC.

## Introduction

Head and neck squamous cell carcinoma (HNSCC) is the sixth most common cancer worldwide (1). Due to late symptomatic illness, patients often present with locally advanced tumors, lymph node and distant metastasis. Although new treatments were established in the past years, the mortality remains high and relapses occur often (2). The lack of tumor-specific markers emphasizes the need of new diagnostic tools for early detection, disease surveillance, and therapy monitoring of HNSCC patients.

Exosomes strongly contribute to an immunosuppressive tumor microenvironment (TME) and play a prominent role in immune evasion (3, 4). Exosomes, the smallest of extracellular vesicles, are generated in the endosomal compartment of the cell and secreted into the extracellular space via exocytosis (5). They are released by all cell types and mediate intercellular communication in different physiological and pathophysiological settings by delivery of their cargo consisting of proteins, RNA, DNA and lipids (6, 7). As they resemble their parental cell to a great extent and freely circulate in all body fluids, exosomes are promising components of non-invasive liquid biopsy (8, 9).

Tumor-derived exosomes (TEX) are highly abundant in plasma of HNSCC patients (10, 11). TEX convey immunosuppressive molecules like PDL1 (11) or cancer stem cell-associated antigens like CD44v3 (12) and suppress the function of lymphocytes (10). Their ability to metabolize exogenous adenosine triphosphate (ATP) into adenosine due to the presence of enzymatically active ectonucleotidases CD39 and CD73 alters immune cell functions in the TME (13). TEX from plasma activate regulatory T cells and thereby reduce anti-tumor immune response (14–16). Previous studies showed that the composition and cargo of TEX correlates with disease stage, tumor activity and progression, assigning them great potential as biomarkers for HNSCC (11, 17–20). Additionally, exosomal miRNAs from plasma or serum show promising disease-discriminatory potential in oral (21) and laryngeal cancer (22).

However, only few studies were carried out to investigate saliva-derived exosomes as potential non-invasive liquid biomarkers. In contrast to plasma, saliva samples are not routinely used in clinical diagnostic although saliva is an easy-to-handle biofluid with good accessibility and an even less invasive collection. Since exosomes are secreted by exocytosis (23), it is most likely that the various cell types lining the upper aerodigestive tract, healthy and malignant, secrete exosomes directly into saliva. TEX resembling a distant tumor reach salivary glands via circulation and can change the composition of salivary exosomes as described for lung (24, 25), pancreatic (26, 27), and breast cancer (28). Additionally, proteomic changes in whole saliva and saliva-derived exosomes were observed in oral cancer patients (29) and exosomal miRNAs from saliva could differentiate between healthy individuals and patients with oral cancer (30–32). Therefore, saliva may be a suitable biofluid for screening programs or early detection of high-risk patients and therapy monitoring in HNSCC.

In this study, we investigate the immunomodulatory properties of saliva-derived exosomes from HNSCC patients regarding their protein and miRNA cargo as well as their functional effects on lymphocytes. This could lead to a better understanding of the potential of saliva-derived exosomes as liquid biopsy in clinical practice.

## Materials and Methods

### Sample Collection

Saliva samples were obtained from newly diagnosed, treatment-naïve HNSCC patients with histologically confirmed tumors seen at the ENT department of Ulm University Hospital as well as from healthy donors (HD) in agreement with the local ethics committee (Votum #90/15). Each patient provided informed consent. The lab examiners were not blinded to the source of the material analyzed. Tables 1, 2 show clinicopathological data of enrolled patients.

**Table 1:** Clinicopathological data of HNSCC patients enrolled in this study.

**Characteristics**	**Patients (*n* = 21)**	
	***n***	**%**
**Age (years)**		
≤ 63	12	57.2
>63	9	43.8
(Range: 49–79)		
**Gender**		
Male	18	85.7
Female	3	14.3
**Primary tumor site**		
Oral cavity	4	19
Oropharynx	11	52.4
HPV positive	4	36
HPV negative	5	46
Status unknown	2	18
Hypopharynx	2	9.6
Larynx	4	19
**Tumor stage**		
T1	4	19
T2	7	33.3
T3	3	14.3
T4	7	33.3
**Nodal status**		
N0	6	28
N+	15	71
**Distant metastasis**		
M0	20	95.2
M+	1	4.8
**UICC stage**		
I	3	14.3
II	4	19
III	3	14.3
IV	11	52.4
**Alcohol/Tobacco**		
Yes	20	95.2
No	1	4.8

*HPV, Human papillomavirus; UICC, Union for International Cancer Control*.

**Table 2:** Clinicopathological data of healthy donors enrolled in this study.

**Characteristics**	**Patients (*n* = 12)**	
	***n***	**%**
**Age (years)**		
≤ 63	9	75
>63	3	25
(Range: 51–71)		
**Gender**		
Male	8	66.7
Female	4	33.3
**Alcohol/Tobacco**		
Yes	6	50
No	4	33.3
Not available	2	16.7

Saliva was collected using Salivette^®^ plain cotton swabs (Sarstedt, Nürmbrecht, Germany, 51.1534) between 07:00 and 10:00 a.m. Patients did not eat, drink or perform dental hygiene for at least 1 h before collection to reduce contamination. The probands sequentially chewed six swabs for 1 min until they were soaked with saliva. Samples were immediately put on ice during the collection process. The swabs were centrifuged at 1,000 × g for 2 min to gather the saliva in the reservoir. The obtained saliva of each sample was pooled to ensure a homogenous mix for each patient. The resulting saliva specimens were stored in aliquots at −80°C until further use.

### Exosome Isolation by Ultracentrifugation

Freshly thawed saliva was centrifuged at 3,000 × g for 10 min at 4°C and 10,000 × g for 30 min at 4°C to remove cell debris and larger vesicles. The supernatants were diluted 1:1 with phosphate-buffered saline (PBS, Gibco, Carlsbad, CA, USA, 14190-094) to reduce viscosity, followed by filtration through 0.22 μm syringe-filters (Millipore, Burlington, MA, USA, SLGPO33RB). The samples were then centrifuged at 120,000 × g for 3 h at °C. The exosome pellets were resuspended in 1 mL PBS and stored at 4°C. The exosome solutions were used for further analysis within 5 days.

Exosomes from plasma were isolated by size exclusion chromatography as previously described (33).

### BCA and Exosome Concentration

Total exosomal and total salivary protein concentration was measured by bicinchoninic acid (BCA) assay (ThermoFisher Scientific, Waltham, MA, USA, 23225) according to the manufacturer's protocol. Exosomes were concentrated using 100 kDa cut-off centrifugal filters (Millipore, UFC5100BK). For Western Blot 30 μg of exosomes in 40 μL PBS, for bead-based flow cytometry and adenosine production 10 μg of exosomes in 100 μL PBS and for functional assays 30 μg of exosomes in 50 μL PBS were used.

### Characterization of Exosomes

Exosome characterization was performed according to the minimal information for studies of extracellular vesicles (MISEV) 2018 guidelines for the definition of extracellular vesicles (34). Transmission electron microscopy (TEM) was used to analyze the size and morphology of the isolated vesicles, and nanoparticle tracking analysis (NTA) for size and particle counting. Western Blot was used to confirm the cellular origin of the vesicles by detecting the endosomal marker TSG101, and the exosome-associated tetraspanins CD9 and CD63. All methods were routinely used and described in detail in our previous publication (20).

### CD63 Immune Capture and On-Bead Flow Cytometry of Exosomes

The immune capture and on-bead flow cytometry was performed as described previously (19, 35). Briefly, exosomes were captured on ExoCap Streptavidin magnetic beads (MBL Life Science, Woburn, MA, USA, MEX-SA) with biotin-labeled anti-CD63 (BioLegend, San Diego, CA, USA, 353018, RRID:AB_2561676). The resulting bead/anti-CD63Ab/exosome complexes were stained with fluorophore-conjugated antibodies. The following detection antibodies and appropriate isotype controls were used: anti-CD39-FITC (328206, RRID:AB_940425), anti-mouse-IgG1-FITC (400110, RRID:AB_2861401), anti-CD73-APC (344006, RIDD:AB_1877157), anti-mouse-IgG1-APC (400120, RRID:AB_2888687), anti-FasL-PE (306407, RIDD:AB_2100664), anti-mouse-IgG1-PE (400112, RRID:AB_2847829) from BioLegend; anti-PDL1-PE (12-5983-42, RRID:AB_11042286), anti-mouse-IgG1-PE (12-4714-42, RRID: AB_1944423) from eBioScience (San Diego, CA, USA); anti-OX40L-APC (FAB10541A, RRID:AB_10642181), anti-mouse-IgG1-APC (IC002A, RRID:AB_357239), anti-CD44v3-APC (FAB5088A, RRID:AB_2076584), anti-PDL2-APC (FAB1224A, RRID:AB_2161997) and anti-mouse-IgG2b-APC (IC0041A, RRID:AB_357246) from R&D Systems (Minneapolis, MN, USA); anti-EpCAM-PE (ab112068, RRID:AB_10861805), anti-mouse-IgG1-PE (ab91357, RRID:AB_2888649) from abcam (Cambridge, UK); anti-CD45-PeCY7 (IM3548, RRID:AB_1575969), anti-mouse-IgG1-PECy7 (737662) from Beckman Coulter (Brea, CA, USA). After staining, the complexes were washed twice with PBS and resuspended in 300 μL PBS for flow cytometry. Detection was performed using a Gallios flow cytometer with Kaluza 1.0 software (Beckman Coulter). Data are presented as relative fluorescent intensity (RFI), which equals the mean fluorescence intensity of the stained sample divided by the mean fluorescence intensity of the isotype control.

### CD69 Induction on CD8^+^ T Cells

CD69 induction on CD8^+^ T cells was performed as previously described (17). CD8^+^ T cells were isolated from peripheral mononuclear cells (PBMCs) from a healthy donor's buffy coat (obtained from DRK Ulm, Helmholtzstraße 10, 89081 Ulm) using the human CD8^+^ T Cell Isolation Kit (Miltenyi Biotec, Bergisch Gladbach, Germany, 130-096-495) according to manufacturer's instruction. CD8^+^ T cells were activated with 25 μl/mL ImmunoCult™ Human CD3/CD28 T Cell Activator (Stemcell Technologies, Vancouver, Canada, 10971) and 150 U/mL recombinant human IL2 (R&D Systems, 202-IL-010) in RPMI 1640 medium (Gibco, 21875-034) supplemented with 10% exosome-depleted fetal bovine serum (FBS, Gibco, A2720801) and 1% penicillin-streptomycin (PAN-Biotech, Aidenbach, Germany, P06-07100). CD8^+^ T cells (200 000 cells in 150 μl) were seeded in 96-well plates and incubated at 37 °C for 6 h. Saliva-derived exosomes from HNSCC patients and healthy donors or plasma-derived exosomes from HNSCC patients were added and incubated for another 16 h. Activated T cells were stained with anti-CD69-FITC antibody (BD, Franklin Lakes, NJ, USA, 555530, RRID:AB_395915) and its expression levels were measured on a Gallios flow cytometer.

### Carboxyfluorescein Succinimidyl Ester (CFSE) Assay of CD4^+^ T Cells

The method was described before in previous publications (10). Briefly, CD4^+^ T cells were isolated from PBMCs of healthy donors by use of the human CD4^+^ T Cell Isolation Kit (Miltenyi Biotec, Bergisch Gladbach, Germany, 130-096-533) according to the manufacturer's instruction. For CFSE staining, CD4^+^ T cells were incubated with 5 μM CellTrace^TM^ CFSE (Thermo Fisher Scientific, Waltham, MA, USA, C34554) at 37°C for 20 min in the dark, according to manufacturer's instructions. CD4^+^ T cells were activated as described above and co-incubated after 24 h with 30 μg salivary exosomes for 4 d. As a positive control, plasma-derived exosomes from HNSCC patients were used (10). Proliferation of CD4^+^ T cells was measured by flow cytometry.

### Adenosine Production by Saliva-Derived Exosomes

For measurement of adenosine production by saliva-derived exosomes, they were incubated with 20 μM ATP (Sigma Aldrich, St. Louis, MO, USA, A2383) for 1 h at 37°C. As controls, exosomes without ATP or ATP only were used. All samples were centrifuged for 2 min at 6,000 × g. Supernatants were boiled for 2 min at 95°C and stored at −80 °C until further use. Levels of ATP, 5' adenosine monophosphate (AMP), adenosine, and their metabolites were measured by mass spectrometry as previously described (15).

### Exosomal RNA Isolation, Quality Control, and Quantification

The miRNeasy Micro Kit (Qiagen, 217084) was used for total RNA isolation according to the manufacturer's instructions. Quality and amount of exosomal RNA were assessed by Agilent 2100 Bioanalyzer (Agilent Technologies) and a Qubit Fluorometer (Thermo Fisher Scientific). Exosomal RNA was stored at −80°C until further processing.

### NanoString miRNA Profiling of Exosomes

miRNA profiling was performed using the nCounter^®^ SPRINT system (Nanostring Technologies) at the nCounter^®^ Core Facility of the University of Heidelberg, Germany. Total exosomal RNA was applied to the Human v3 miRNA Assay covering the measurement of 827 human miRNAs. The assay was performed with 500 pg of total exosomal RNA according to the manufacturer's instructions.

### Computational and Statistical Analysis

Plots were generated in GraphPad Prism (version 9, GraphPad Software, San Diego, CA, RRID:SCR_002798). Box-and-whisker blots show the median, the interquartile range (25–75%), and the range. Comparisons between groups were analyzed by Mann-Whitney test for independent samples and p ≤ 0.05 was considered statistically significant. The Volcano plot was generated using the log2 expression ratio of each miRNA and the negative log10 of the *p*-value.

miRNA expression data were analyzed using the nSolver 4.0 software. Raw counts of individual miRNAs were normalized using the positive ligation controls. Then, data analysis was performed in R (4.0.2, RRID:SCR_001905) using the IDE Rstudio (1.3.1056). Ggplot2 (3.3.2) was used for data visualization of heatmaps and Uniform Manifold Approximation and Projection (UMAP) plots. Clustering in the heatmaps was done using hierarchical clustering within the pheatmap (1.0.12) package. UMAP dimensionality reduction was performed using the package uwot (0.1.8), with the default parameters (except: n_neighbors = n/2). For computation of distance matrices between samples, Euclidian distance was used.

The Venn diagram was generated using InteractiVenn (http://www.interactivenn.net/) (36).

Pathway and network analysis were performed using Ingenuity Pathway Analysis (IPA, Qiagen, RRID:SCR_008653).

“Hsa” has been removed from the miRNA names throughout the manuscript for simplification.

## Results

### Study Population

Table 1 shows the clinicopathological characteristics of HNSCC patients whose saliva was used for this study. The mean age was 63 years with a range from 49 to 79 years. The majority of patients (86 %) was male. In 19 % the tumor was located in the oral cavity, in 62% in the pharynx, and 19 % were localized in the larynx. Four oropharyngeal tumors were human papillomavirus (HPV) positive (33 %) and five HPV-negative (42%). In two patients, HPV status was not determined. Forty-eight percent of patients presented with advanced local tumor stage (T3/4) and 71 % had lymph node metastasis. One patient had a distant metastasis in the lung. According to Union for International Cancer Control (UICC), 33 % of patients were assigned to low/non-advanced stage and 67 % to high/advanced stage.

### Characterization of Saliva-Derived Exosomes

**Figure 1:**
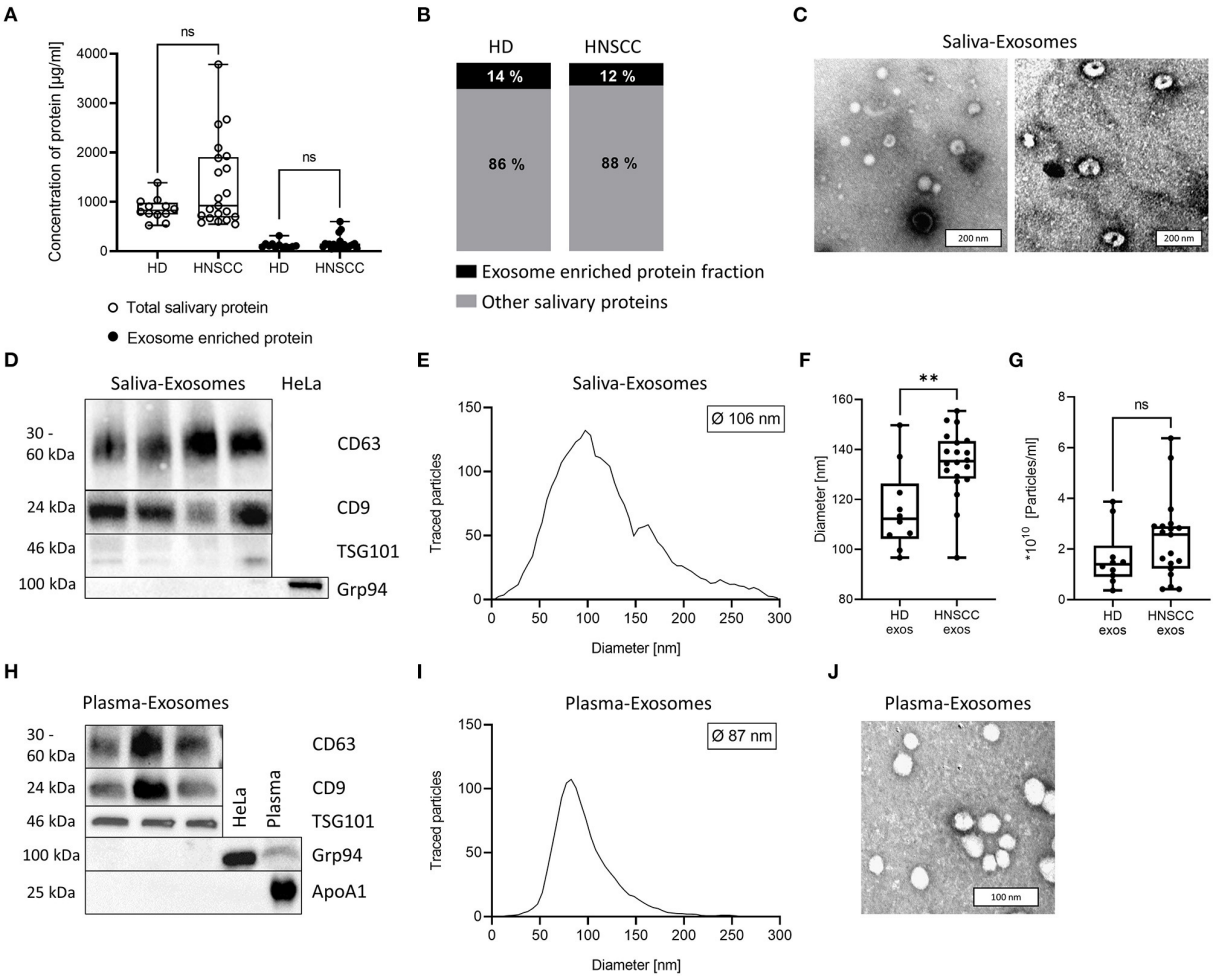
Characterization of exosomes isolated from human saliva and plasma. **(A)** Total salivary protein and exosome enriched protein obtained from saliva of healthy donors (HD; *n* = 12) and head and neck squamous cell carcinoma (HNSCC) patients (*n* = 21). **(B)** The ratio between exosome enriched protein and total saliva protein was determined for each sample and average percentage is shown for HD and HNSCC patients. **(C)** Representative transmission electron microscopy (TEM) images of exosomes derived from saliva of one HNSCC patient (left) or HD (right). **(D)** Western blot for detection of exosomal markers CD63, CD9, TSG101 and the cellular marker Grp94 in saliva-derived exosome preparations. HeLa-cell-lysate was used as a positive control for Grp94. **(E)** Nanoparticle tracking analysis (NTA) of saliva-derived exosomes. The histogram shows a representative size distribution of saliva-derived vesicles with a median diameter of 106 nm. **(F)** Exosomes from saliva of HNSCC patients showed a higher median diameter (*n* = 20, 135 nm) compared to exosomes from saliva of HD (*n* = 10; median diameter 116 nm). **(G)** Concentration of detected vesicles/ml was analyzed by NTA and compared between HD (*n* = 10) and HNSCC patients (*n* = 19). **(H)** Western blot for detection of exosomal markers CD63, CD9, TSG101, the cellular marker Grp94 and contaminant apolipoprotein ApoA1 in plasma-derived exosome preparations. HeLa-cell-lysate and plasma were used as a positive control for Grp94 and ApoA1, respectively. **(I)** NTA of plasma-derived exosomes. The histogram shows a representative size distribution of plasma-derived vesicles with a median diameter of 87 nm. **(J)** Representative TEM image of HNSCC patient's plasma-derived exosomes. **(A,F,G)** Results are plotted as box-and-whisker blots representing the median value, the 25^th^ and 75^th^ quartiles and the range. *P*-values were determined by Mann–Whitney test, **p ≤ 0.01, ***p ≤ 0.001, ns, not significant.

Protein concentration of total salivary and exosome-enriched samples were measured and compared between HD and HNSCC. No significant difference was observed (Figure 1A). Furthermore, the exosome-enriched protein fraction in saliva did not differ between HD (14 %) and HNSCC patients (12 %) (Figure 1B). In TEM, exosomes isolated from saliva showed the characteristic cup-shaped morphology (Figure 1C). Western blot analysis of saliva-derived exosomes confirmed the presence of the tetraspanins CD63 and CD9 and the endosomal marker TSG101, while the cellular marker Grp94 was not detected (Figure 1D). The diameter ranged from 20 to 300 nm with a median diameter of 106 nm (Figure 1E). Saliva-derived exosomes from HNSCC patients had a significantly higher median diameter compared to HD saliva-derived exosomes (Figure 1F), whereas the particle number was only slightly elevated (Figure 1G). No significant differences in particle diameter and number were observed dependent on tumor site or UICC stage (Supplementary Figure 1).

Plasma-derived exosomes from HNSCC, which were used as controls for functional assays, were also characterized as recommended by the MISEV 2018 guidelines. They were positive for tetraspanins CD63 and CD9 as well as TSG101 but negative for Grp94 and the contaminant apolipoprotein ApoA1 (Figure 1H). Further, their diameter ranged from 30-200 nm with a median diameter of 87 nm (Figure 1I), and they showed characteristic morphology (Figure 1J).

### Saliva-Derived, CD63-Captured Exosomes From HNSCC Patients Carry High Amounts of Surface CD44v3, PDL1 and CD39

**Figure 2:**
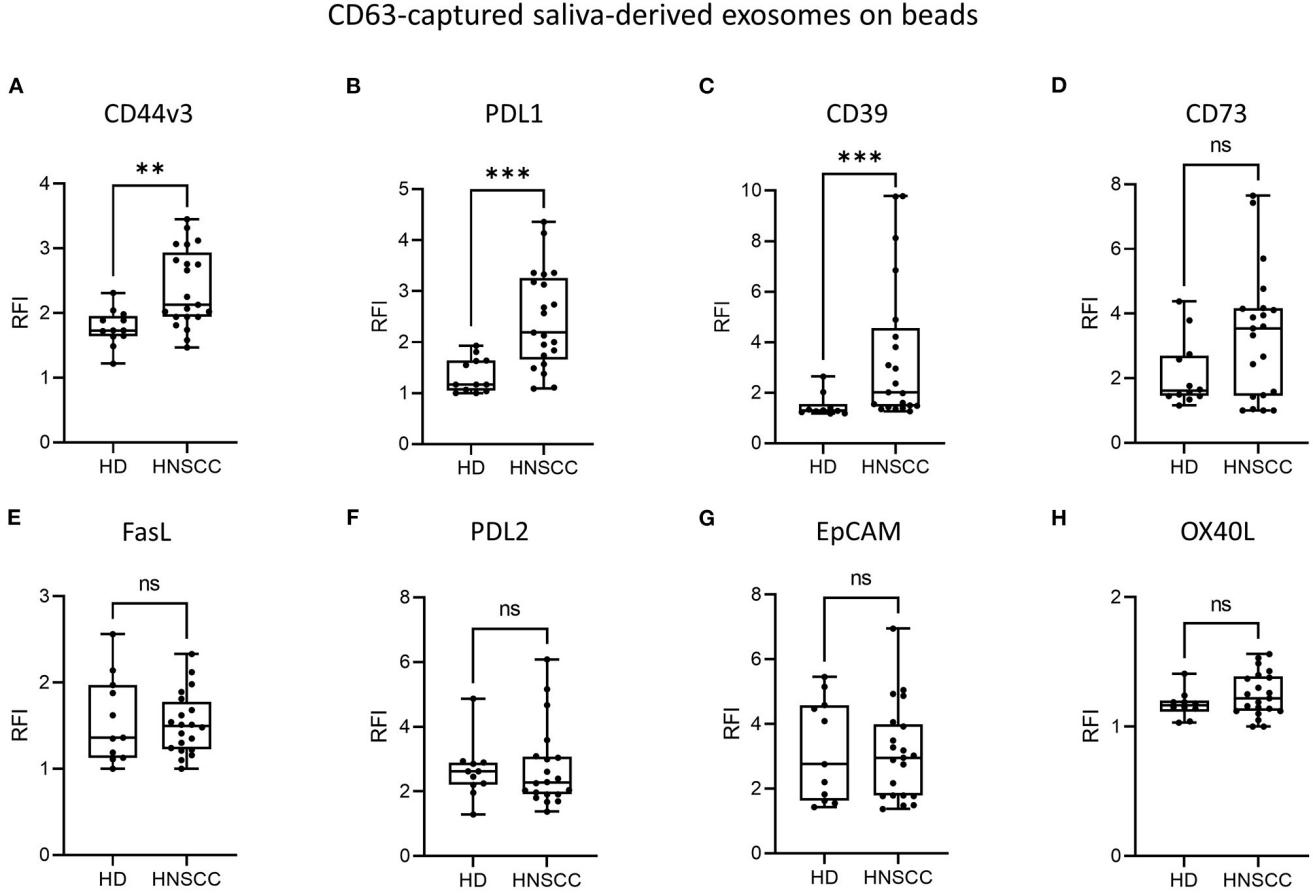
Surface levels of different antigens on saliva-derived, CD63-captured exosomes. Exosomes isolated from saliva of HD (*n* = 12) and HNSCC patients (*n* = 22) were stained for **(A)** CD44v3, **(B)** PDL1, **(C)** CD39, **(D)** CD73, **(E)** FasL, **(F)** PDL2, **(G)** EpCAM and **(H)** OX40L using capture with CD63 antibodies and subsequent bead-based flow cytometry. Surface levels are shown as relative fluorescence intensity (RFI) compared to isotype controls. Saliva-derived exosomes from HNSCC patients showed significantly higher surface values of CD44v3 **(A)**, PDL1 **(B)** and CD39 **(C)** compared to saliva-derived HD exosomes. Results are plotted as box-and-whisker blots representing the median value, the 25^th^ and 75^th^ quartiles and the range. *P*-values were determined by Mann-Whitney test, **p ≤ 0.01, ***p ≤ 0.001, ns, not significant.

To evaluate the immunomodulatory characteristics of saliva-derived exosomes from HNSCC patients, levels of immunosuppressive molecules and squamous cell carcinoma associated surface antigens were measured by on-bead flow cytometry, using capture with CD63 antibodies. Both HD and HNSCC saliva-derived exosomes were positive for CD44v3, while RFI values were significantly higher (*p* < 0.01) for exosomes from HNSCC patients compared to HD (Figure 2A). RFI values for the co-inhibitory immune checkpoint molecule PDL1 were significantly higher (*p* < 0.001) on saliva-derived exosomes from HNSCC patients compared to HD (Figure 2B). Furthermore, saliva-derived exosomes from HNSCC patients showed higher RFI values for CD39 (*p* < 0.001, Figure 2C). The surface RFI values of CD73 were heterogeneous in both groups and showed a trend toward higher values on saliva-derived exosomes from HNSCC patients (Figure 2D). Surface values of FasL, PDL2, EpCAM and OX40L did not differ between saliva-derived exosomes from HD and HNSCC patients (Figures 2E–H). Further, no significant differences in the RFI values of analyzed antigens were observed according to tumor site (Supplementary Figure 2) or UICC stage (Supplementary Figure 3).

### No Functional Effects on CD8^+^ T Cells and CD4^+^ T Cells by Saliva-Derived Exosomes

**Figure 3:**
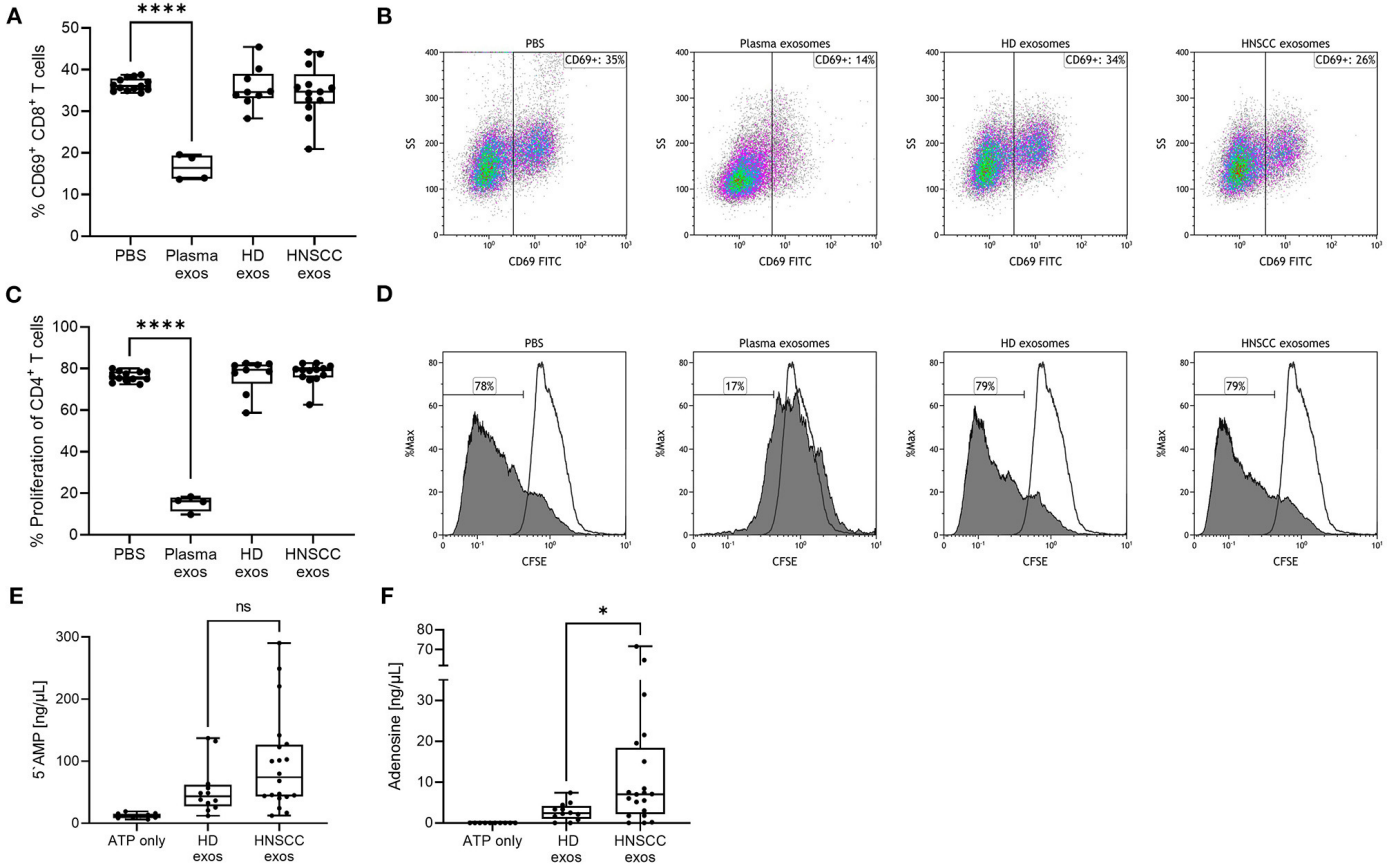
Functional effects of saliva-derived exosomes on activated CD8^+^ T cells, proliferating CD4^+^ T cells and ATP metabolism. **(A,B)** Activated CD8^+^ T cells were co-incubated with saliva-derived exosomes from HD (*n* = 9) and HNSCC patients (*n* = 13) for 16 h. CD69 expression of T cells was measured by flow cytometry. **(B)** shows representative plots for each condition. **(C,D)** CD4^+^ T cells were co-incubated with saliva-derived exosomes from the same HD (n = 9) and HNSCC patients (*n* = 13) for 4 d. Proliferation was evaluated by CFSE assay and flow cytometry. **(D)** shows representative histograms for each condition. Filled histograms represent proliferated cells and framed histograms the unstimulated parental generation. For **(A–D)**, plasma-derived exosomes obtained from HNSCC patients (*n* = 4) were included as positive controls. **(E)** 5'AMP and **(F)** adenosine levels were measured by mass spectrometry upon incubating saliva-derived exosomes of HD (*n* = 12) and HNSCC patients (*n* = 20) with exogenous ATP. Results in **(A,C,E,F)** are plotted as box-and-whisker blots representing the median value, the 25^th^ and 75^th^ quartiles and the range. P-values were determined by Mann–Whitney test, *p ≤ 0.05, ****p ≤ 0.0001, ns, not significant.

Highly immunosuppressive effects of plasma-derived exosomes from HNSCC patients on T cells have been described in previous publications (11, 15, 18, 19). To investigate whether saliva-derived exosomes exhibit a similar effect on T cells, activated CD8^+^ or CD4^+^ T cells were co-incubated with saliva-derived exosomes from HNSCC patients and HD as well as plasma-derived exosomes from HNSCC patients as a positive control. CD69 expression on CD8^+^ T cells was analyzed by flow cytometry as a marker of T cell activation. Upon stimulation, around 36 % of CD8^+^ T cells showed expression of CD69 (Figure 3A). As expected, a high and significant suppression of CD8^+^ T cell activation was observed upon incubation with plasma-derived exosomes (CD69 expression of 16 %). CD69 expression was not affected by saliva-derived exosomes from HNSCC patients (mean CD69 expression of 35 %) nor HD exosomes (mean CD69 expression of 36 %) (Figure 3A). Representative density plots are shown in Figure 3B.

Next, the effect on CD4^+^ T cell proliferation was evaluated using a CFSE assay. Stimulated CD4^+^ T cells showed a proliferation rate of around 77% (Figure 3C). The proliferation rate of CD4^+^ T cells was significantly suppressed to 15% upon incubation with plasma-derived exosomes from HNSCC patients. When incubated with saliva-derived exosomes from both HD and HNSCC patients, CD4^+^ T cell proliferation was not affected (Figure 3C). Representative histograms are shown in Figure 3D.

Tumor site or UICC stage did not show differential effects on CD8^+^ nor CD4^+^ T cells (Supplementary Figure 4).

### Significant Production of Adenosine by Saliva-Derived Exosomes From HNSCC Patients

Plasma-derived exosomes of HNSCC patients were found to be potent producers of immunosuppressive adenosine (14, 15). Since saliva-derived exosomes showed substantial amounts of CD39 and CD73 on their surface, we examined their potential to produce immunomodulating metabolites of ATP. Both saliva-derived exosomes from HD and HNSCC patients were able to independently produce 5'AMP and adenosine (Figures 3E,F). Exosomes from HNSCC patients produced higher 5'AMP levels compared to exosomes from HD, although non-significant (*p* = 0.146, Figure 3E). However, production of immune suppressive adenosine was significantly higher (*p* < 0.05) in exosomes from HNSCC patients compared to HD (Figure 3F). Tumor site or UICC stage did not differentially influence the production of 5‘AMP or adenosine (Supplementary Figure 4).

### Saliva-Derived Exosomes Are Enriched in TEX and Depleted in Non-TEX

**Figure 4:**
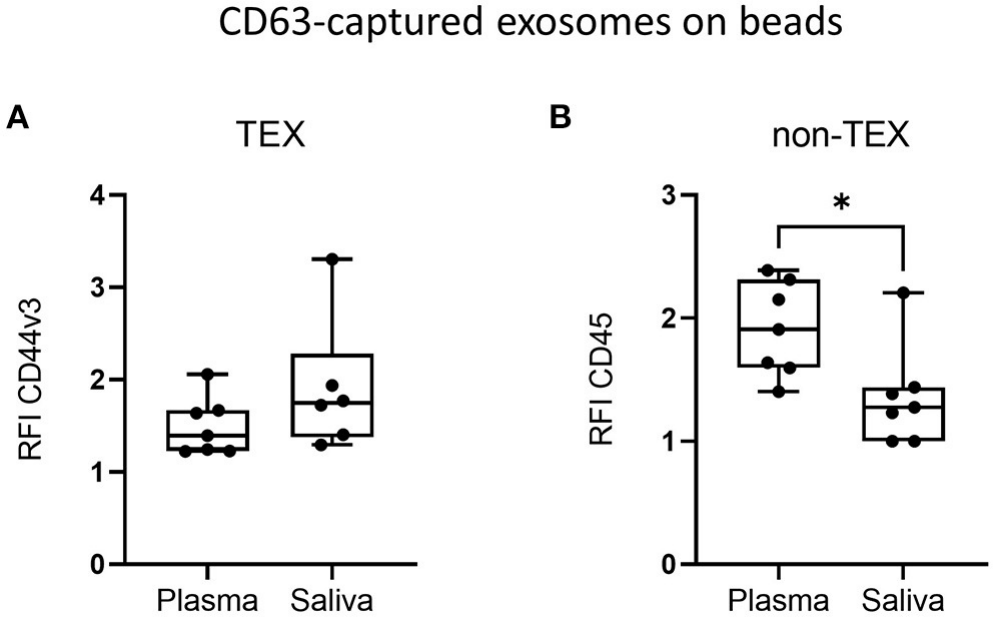
Tumor-derived exosomes (TEX) and non-TEX amounts in total exosome populations from saliva. Exosomes isolated from plasma and saliva of HD and HNSCC patients (n = 7) were stained for **(A)** CD44v3 (TEX) and **(B)** CD45 (non-TEX) using bead-based flow cytometry. Surface levels are shown as RFI compared to isotype controls. Saliva had significantly lower amounts of CD45^+^ exosomes compared to plasma **(B)**. Results are plotted as box-and-whisker blots representing the median value, the 25^th^ and 75^th^ quartiles and the range. P-values were determined by Mann–Whitney test, *p ≤ 0.05.

To identify the origin of saliva-derived exosomes and the relative abundance of TEX and non-TEX among the total exosome population, the amount of CD44v3^+^ (TEX enriched) and CD45^+^ (non-TEX enriched) exosomes were compared between plasma and saliva. Saliva showed slightly higher amounts of CD44v3^+^ exosomes (Figure 4A) and significantly lower amounts of CD45^+^ exosomes (Figure 4B) compared to plasma, indicating a low contribution of hematopoietic cell-derived exosomes to the total exosome population in saliva.

### miRNA Profiling of Saliva-Derived Exosomes

**Figure 5:**
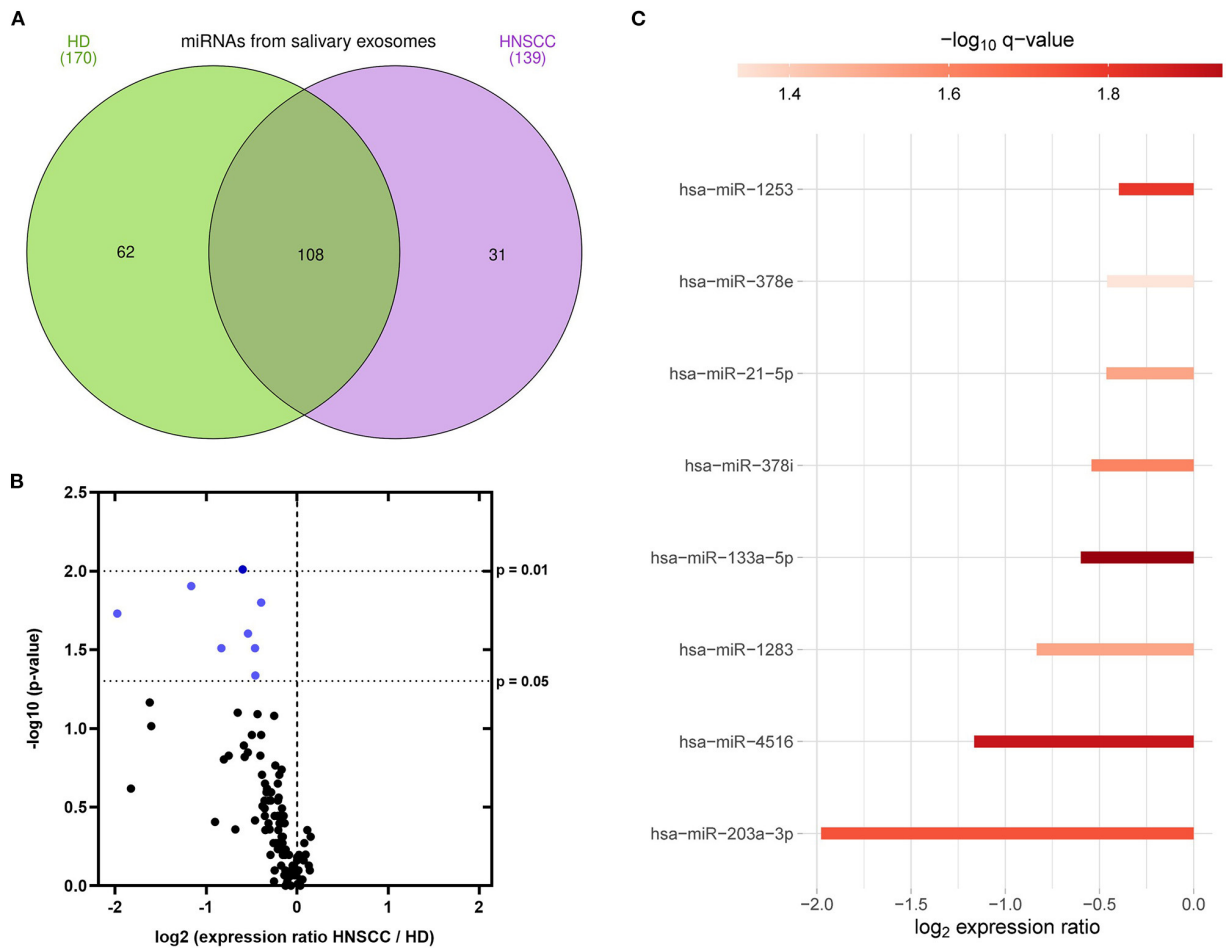
miRNA profiling of saliva-derived exosomes. **(A)** Venn diagram of miRNAs detected in saliva-derived exosomes of HD (green, *n* = 7) and HNSCC patients (purple, *n* = 14), created with InteractiVenn (36). **(B)** Volcano plot showing the log2 expression ratio of 101 miRNAs overlapping between saliva-derived exosomes of HD and HNSCC patients. Each dot represents one miRNA. miRNAs at x > 0 are upregulated in HNSCC saliva-derived exosomes, while miRNAs at x < 0 are downregulated in HNSCC saliva-derived exosomes compared to HD. miRNAs in blue are significantly downregulated (p ≤ 0.05) in HNSCC saliva-derived exosomes compared to HD. **(C)** Waterfall plot of significant miRNAs from **(B)** showing the log2 fold change difference between HD and HNSCC saliva-derived exosomes. Red color indicates the negative logarithm of the *p*-value (the stronger the color, the more significant).

To investigate the miRNA cargo of saliva-derived exosomes, miRNA profiling was performed and compared between HD and HNSCC patients. A total of 170 exosomal miRNAs were detected in saliva of HD, while 139 exosomal miRNAs were detected in saliva of HNSCC patients (Figure 5A). 62 miRNAs were exclusively present in saliva-derived exosomes from HD, 31 in exosomes from HNSCC patients and 108 were overlapping between the two groups (Figure 5A, Supplemenatry Table 1). A majority (86 %) of the overlapping saliva-derived exosomal miRNAs showed lower levels in HNSCC patients compared to HD (Figure 5B, Supplementary Table 2). 8 miRNAs had significantly lower expression ratios (marked in blue in Figure 5B) with miR-203a-3p showing the greatest fold change and miR-133a-5p having the highest significance (Figure 5C, Supplementary Table 2).

**Figure 6:**
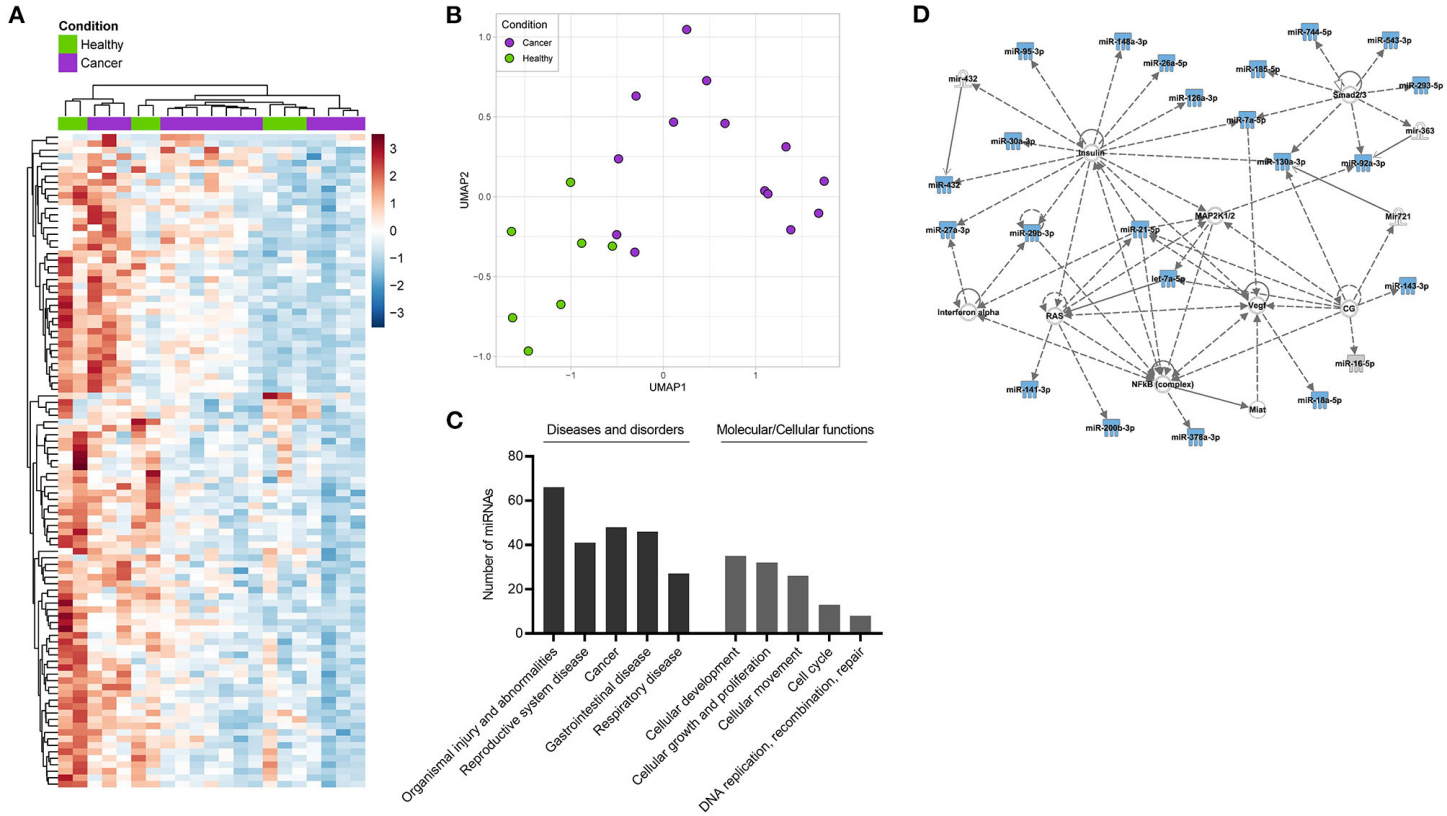
Clustering and pathway analysis of miRNAs from saliva-derived exosomes. **(A)** Unsupervised hierarchical clustering heatmap and **(B)** Uniform manifold approximation and projection (UMAP) visualizations of saliva-derived miRNAs which were HD- or HNSCC-exclusive or overlapped with significant difference between the two groups (total of 101 miRNAs). **(C,D)** Ingenuity pathway analysis (IPA) of the same miRNAs. **(C)** Shows diseases and biological functions most significantly associated with the identified miRNAs. Categories within a group are shown in increasing order of *p*-value. **(D)** Shows the highest ranked network to which identified miRNAs (blue symbols) contribute. Solid lines indicate direct interaction, dashed lines indicate indirect relation.

Following, HD- and HNSCC-exclusive, as well as overlapping saliva-derived exosomal miRNAs with significant difference between both groups (total of 101 miRNAs), were chosen for clustering, pathway and network analysis. Unsupervised hierarchical clustering and UMAP analysis were able to group samples according to “Healthy” and “Cancer” based on their saliva-derived exosomal miRNA profile (Figures 6A,B). Yet, the clustering was not complete, as some HD were close to a cancer-like signature. Pathway and network analysis by IPA revealed a biological role of saliva-derived exosomal miRNAs in “organismal injury and abnormalities” and “cancer” (Figure 6C). Molecular functions were associated with cellular development, growth, proliferation, and movement as well as cell cycle and DNA replication. Furthermore, saliva-derived exosomal miRNAs were found to be involved in RAS/MAPK, NF-κB complex, Smad2/3, and IFN-α signaling (Figure 6D).

## Discussion

Despite the fact of several beneficial properties of saliva as liquid biopsy, this body fluid is not widely used in clinical diagnostics of HNSCC. Compared to blood or tissue biopsies, the non-invasive collection of saliva reduces patient's discomfort and pain and can also be acquired from patients with poor veins or anemia. Repetitive saliva collection within screening programs during disease surveillance is even feasible by patients themselves thereby reducing the workload of healthcare professionals. Still, saliva sampling can be impeding in patients suffering from xerostomia due to radiotherapy, surgery of the salivary glands, or psychological stress. Salivary flow and composition are further affected by circadian rhythm and stress (37), food uptake, gingival bleeding, or alcohol and tobacco consumption (38). Even alcohol-/tobacco-induced changes in the RNA profile of airway epithelial cell EVs have been described (39), but the influence of tobacco/alcohol on EV composition in oral cancer is rarely studied (40). To minimize variability and confounders, we considered these factors by standardized collection of saliva samples in defined time frames prior to food uptake. All HNSCC patients except one consumed alcohol and/or tobacco. Among healthy donors, we did not identify significant differences in any result when comparing groups with and without alcohol and tobacco consumption, indicating no dependence of analyzed parameters on alcohol/tobacco.

Exosomes in saliva are thought to provide a less complex, stable, and clinically relevant basis for disease detection (41). Our previous studies revealed the potential of plasma-derived exosomes as promising biomarkers for disease activity and tumor stage in HNSCC (11, 12, 15, 18, 20). To establish alternative, possibly synergistic diagnostic tools for the detection of HNSCC, we here analyzed the biological and functional properties of saliva-derived exosomes. We evaluated saliva-derived exosomes from HNSCC patients and healthy individuals regarding their protein and miRNA cargo and their functional properties.

Saliva-derived exosomes from HNSCC patients, compared to HD, had a higher median diameter, which is consistent with earlier studies comparing morphologies of saliva-derived exosomes in oral cancer (30, 42–44). Although they also reported an increased vesicle concentration in saliva of oral cancer patients, we and Gai et al. (30) could only observe a trend toward higher exosome levels in HNSCC or OSCC patients, respectively. This can be due to the different quantification method by atomic force microscopy (43) or the use of pooled oral fluid samples (42). Our data indicate that in HNSCC the composition and biological properties of saliva-derived exosomes - causing an increase in size - seem to be different rather than the quantity of secreted vesicles compared to healthy donors.

Proteomic approaches with saliva-derived extracellular vesicles from oral cancer (29) and lung cancer patients (24) revealed discriminatory potential of saliva-derived extracellular vesicles between “tumor” and “healthy,” suggesting their reflection of the originating tumor. In our previous studies on plasma-derived exosomes, we identified several immune checkpoint molecules to be differentially present on exosomes from HNSCC patients and HD (10–12, 19). To evaluate their qualitative differences on saliva-derived exosomes from HNSCC patients and HD, we compared surface values of both inhibitory (PDL1, PDL2, FasL) and stimulatory immune checkpoint molecules (OX40L). Due to methodological and technical reasons, only CD63-captured exosomes were considered in the awareness that they do not cover the whole exosome populations. Still, PDL1 showed significantly elevated values on saliva-derived exosomes from HNSCC patients. This is of great interest as we previously described both elevated values on plasma-derived exosomes from HNSCC patients with correlation to disease activity (11) and the ability of plasma-derived exosomal PDL1 to discriminate between responders and non-responders to conventional therapy (17). Yu et al. observed elevated levels of PDL1 mRNA in saliva-derived exosomes in patients with periodontitis, suggesting a role of saliva-derived exosomal PDL1 in inflammatory response (45). Saliva-derived exosomal CD44v3, a glycoprotein commonly overexpressed in HNSCC tissues (46, 47) and involved in tumor progression (46, 48), and the ectonucleotidase CD39, an enzyme involved in adenosine production (49), were significantly elevated in HNSCC patients. The epithelial cell adhesion molecule EpCAM was not altered between saliva-derived exosomes from HNSCC patients and HD, which is not surprising given its heterogeneous expression in HNSCC (50–52). We reported earlier that especially CD44v3^+^ plasma-derived exosomes, which are enriched in TEX, carry PDL1 and other immunosuppressive molecules (12). Further, we have shown that TEX carry enzymatically active CD39 and CD73 thus being able to independently produce adenosine in the presence of exogenous ATP (10, 14, 15, 18). Here, we confirmed the ability to metabolize exogenous ATP into adenosine also for saliva-derived exosomes. This is consistent with the observed higher amount of CD39 on the same exosomes. The enrichment of HNSCC saliva-derived exosomes in CD44v3, PDL1 and CD39 and the production of adenosine indicate that a majority of saliva-derived exosomes are TEX reflecting properties of the immunosuppressive TME.

We investigated the ability of saliva-derived exosomes for T cell inhibition and adenosine production. Surprisingly, compared to plasma-derived exosomes, saliva-derived exosomes from HNSCC patients had no impact on the activity of CD8^+^ T cells nor on the proliferation of CD4^+^ T cells. Yet, saliva-derived exosomes from HNSCC patients were strong independent producers of adenosine. The marginal direct effects of saliva-derived exosomes on T lymphocytes suggest that they rather indirectly influence the TME by supporting the accumulation of adenosine, and/or that they preferably target other cell types for immunomodulation, such as NK cells as reported by Katsiougiannis et al. (27). Our previous studies showed that plasma-derived exosomes from HNSCC patients severely alter lymphocyte activity and function (10, 11). Looking closer, these alterations were not only visible with TEX enriched fractions but a significant amount of suppression was observed with CD45^+^ exosome populations, indicating a high immunosuppressive effect not only from TEX but also hematopoietic cell-derived exosomes with a high synergistic effect (15, 19). In this study, we showed that the majority of saliva-derived exosomes in HNSCC patients originate from tumor cells and not hematopoietic cells, which could be a further explanation for the poor suppression of T cell functions.

Previous studies confirm the abundance of miRNAs in saliva-derived exosomes (53, 54) and even show that the majority of miRNAs in saliva and serum is concentrated in exosomes (55). Exosomal miRNAs from saliva were already described to differentiate patients with oral cancer (30–32) or HNSCC (56) from healthy subjects. We here identified 62 and 31 miRNAs exclusive for saliva-derived exosomes of HD and HNSCC patients, respectively. Their unique abundance in either of the groups already highlights their potential as diagnostic biomarker. In addition, 8 of the overlapping saliva-derived exosomal miRNAs showed significantly differential presence with lower levels in HNSCC patients. These totally 101 miRNA candidates were associated with pathways of carcinogenesis including Ras/MAPK, NF-κB, Smad2/3 and IFNα signaling. Genes involved in Ras/MAPK and NF-κB signaling are commonly altered or mutated in HNSCC (57–60), and INFα promotes an immunosuppressive TME (61). Smad2/3 mediate TGFβ signaling and are thus involved in regulation of tumor progression and metastasis by epithelial to mesenchymal transition (62–64). The association of saliva-derived exosomal miRNAs in these pathways emphasizes their contribution to biogenesis, progression and immunomodulation of HNSCC.

Saliva-derived exosomal miR-133a-5p showed the strongest discriminatory potential (p < 0.01) and was downregulated in HNSCC patients compared to HD. This is in line with previously reported reduced tissue expression and tumorsuppressive properties of miR-133a in esophageal cancer (65, 66) and HNSCC (67). Similarly, saliva-derived exosomal miR-203a-3p revealed the greatest fold change with a strongly reduced abundance in HNSCC patients, and tissue miR-203 has been attributed an anti-proliferative and invasion-suppressive role in esophageal cancer (68, 69), nasopharyngeal carcinoma (70) and HNSCC (71). Although no saliva-derived exosomal miRNAs overlapped between the previously reported candidates (30–32, 56) and our study, nor among the previous studies, our results show other miRNA signatures as promising diagnostic tools for HNSCC detection. The discrepancy to the other publications emphasizes the need for standardized methods for isolation and analysis of exosomes to improve reproducibility and comparability between data obtained from different laboratories.

One limitation of our study is the limited sample size, diminishing statistical power, especially concerning associations with clinical data. We plan to further examine the biomarker potential of promising readouts arising from this study in a follow-up cohort with a greater patient number. Another limitation is that the analysis of CD63-captured exosomes only allows for detection of immunomodulatory antigens on a single exosome subpopulation. In preliminary work, we observed that HNSCC exosomes are strongly and consistently positive for CD63, which is why the capture of exosomes with CD63 was chosen. However, using an antibody cocktail including other tetraspanins, such as CD9 and CD81, would be more accurate to represent surface expressions of a broader exosome population. Yet, troubleshooting is expected to be higher with this approach with increased unspecific antibody-epitope interference.

Our work provides evidence that saliva-derived exosomes from HNSCC patients are enriched in TEX whose cargo and functional profile reflect an immunosuppressive TME. Surface values of CD44v3, PDL1 and CD39, adenosine production and the miRNA cargo of saliva-derived exosomes emerged as discriminators of disease and - upon validation in bigger patient cohorts - imply their potential as liquid biomarkers specific for HNSCC.

## Data Availability Statement

The data presented in the study are deposited in the GEO repository, accession number GSE207030 (https://www.ncbi.nlm.nih.gov/geo/query/acc.cgi?acc=GSE207030).

## Ethics Statement

The studies involving human participants were reviewed and approved by Ethics Committee of Ulm University. The patients/participants provided their written informed consent to participate in this study.

## Author Contributions

M-NT and LH: conceptualization and project administration. VM, LH, and M-NT: data curation and investigation. VM, LH, JE, and RR: formal analysis. M-NT: funding acquisition. M-NT, LH, VM, EJ, BN, and RR: methodology. M-NT, CB, TH, EJ, RL, and BN: resources. VM, LH, and JE: software and visualization. VM and LH: writing—original draft. M-NT, CB, DE, SL, PS, and TH: writing—review and editing. All authors have read and agreed to the published version of the manuscript.

## Funding

This work was supported in part by the German Research Foundation (DFG) to M-NT (Grant number TH 2172/2-1) and the Brigitte und Dr. Konstanze Wegener-Stiftung to M-NT.

## Conflict of Interest

The authors declare that the research was conducted in the absence of any commercial or financial relationships that could be construed as a potential conflict of interest.

## Publisher's Note

All claims expressed in this article are solely those of the authors and do not necessarily represent those of their affiliated organizations, or those of the publisher, the editors and the reviewers. Any product that may be evaluated in this article, or claim that may be made by its manufacturer, is not guaranteed or endorsed by the publisher.
