# Supplementary material for: Cargo and Functional Profile of Saliva-Derived Exosomes Reveal Biomarkers Specific for Head and Neck Cancer
Source: Front Med (Lausanne). 2022 Jul 11;9:904295. doi: 10.3389/fmed.2022.904295 (PMC9309685; doi:10.3389/fmed.2022.904295)
Supplement: Supplementary file 1 [file Data_Sheet_1.docx]

**Supplementary Material for**

**“Cargo and functional profile of saliva-derived exosomes reveal biomarkers specific for Head and Neck Cancer”**

**Supplementary Figure 1: Tumor site and UICC stage did not differentially influence exosome-enriched protein concentration, particle size or particle number.**

Exosome-enriched protein was determined by bicinchoninic acid (BCA) assay, particle size and number by nanoparticle tracking analysis (NTA) of exosomes from n = 21 (BCA) / n = 20 (NTA) HNSCC patients. Oral cavity n = 4, pharynx n = 13, larynx n = 4 (BCA) / n = 3 (NTA). UICC I/II n = 7 (BCA) / n = 6 (NTA), UICC III/IV n = 14.

**Supplementary Figure 2: Tumor site did not differentially influence RFI values of analyzed antigens.** Surface levels of antigens on exosomes from HNSCC patients (n = 21) measured by bead-based flow cytometry using CD63-capture. Results are shown as relative fluorescence intensity (RFI) compared to isotype controls. Oral cavity n = 4, pharynx n = 13, larynx n = 4.

**Supplementary Figure 3: UICC stage did not differentially influence RFI values of analyzed antigens.** Surface levels of antigens on exosomes from HNSCC patients (n = 21) measured by bead-based flow cytometry using CD63-capture. Results are shown as relative fluorescence intensity (RFI) compared to isotype controls. UICC I/II n = 7, UICC III/IV n = 14.

**Supplementary Figure 4: Tumor site and UICC stage did not differentially influence CD8^+^ T cell activity, CD4^+^ T cell proliferation, production of 5`AMP or adenosine.**

CD69 expression on activated CD8^+^ T cells and proliferation of CD4^+^ T cells using CFSE assay were analyzed upon incubation with saliva-derived exosomes from n = 13 HNSCC patients. Oral cavity n = 3, pharynx n = 7, larynx n = 3. UICC I/II n = 6, UICC III/IV n = 7.

5`AMP and adenosine levels measured by mass spectrometry upon incubation saliva-derived exosomes of n = 20 HNSCC patients with exogenous ATP. Oral cavity n = 4, pharynx n = 13, larynx n = 3. UICC I/II n = 6, UICC III/IV n = 14.

**Supplementary Table 1**: Saliva-derived exosomal miRNAs from Venn diagram in Fig. 5A in alphabetical order

| **HD-exclusive miRNAs** | **Overlapping miRNAs** | **HNSCC-exclusive miRNAs** |
| --- | --- | --- |
| let-7g-5p | let-7a-5p | miR-1245b-5p |
| miR-1185-2-3p | let-7b-5p | miR-1257 |
| miR-1249-3p | let-7f-5p | miR-127-3p |
| miR-126-3p | miR-10b-5p | miR-1271-5p |
| miR-141-3p | miR-1197 | miR-128-1-5p |
| miR-143-3p | miR-1246 | miR-208b-3p |
| miR-147a | miR-1253 | miR-219b-3p |
| miR-148a-3p | miR-1255a | miR-3147 |
| miR-15b-5p | miR-1258 | miR-340-5p |
| miR-1827 | miR-125b-5p | miR-370-3p |
| miR-185-5p | miR-1268b | miR-377-3p |
| miR-18a-5p | miR-1272 | miR-432-5p |
| miR-196a-5p | miR-1283 | miR-4455 |
| miR-1976 | miR-1285-5p | miR-4755-5p |
| miR-199a-5p | miR-1290 | miR-501-3p |
| miR-200c-3p | miR-1295a | miR-506-5p |
| miR-211-5p | miR-1296-3p | miR-508-3p |
| miR-23b-3p | miR-1297 | miR-516b-5p |
| miR-25-3p | miR-1305 | miR-517a-3p |
| miR-26b-5p | miR-1323 | miR-518f-3p |
| miR-27a-3p | miR-133a-5p | miR-525-5p |
| miR-27b-3p | miR-134-3p | miR-526a+518c-5p+518d-5p |
| miR-29b-3p | miR-142-3p | miR-548ad-3p |
| miR-301a-3p | miR-148b-3p | miR-549a |
| miR-302b-3p | miR-155-5p | miR-593-3p |
| miR-3074-3p | miR-16-5p | miR-605-5p |
| miR-30a-3p | miR-183-5p | miR-612 |
| miR-320e | miR-186-5p | miR-7-5p |
| miR-337-5p | miR-188-5p | miR-744-5p |
| miR-33b-5p | miR-1972 | miR-936 |
| miR-363-3p | miR-199a-3p+199b-3p | miR-95-3p |
| miR-369-3p | miR-200b-3p |  |
| miR-371a-5p | miR-203a-3p |  |
| miR-376c-5p | miR-205-5p |  |
| miR-378d | miR-2053 |  |
| miR-451a | miR-21-5p |  |
| miR-4536-3p | miR-2110 |  |
| miR-4536-5p | miR-223-3p |  |
| miR-454-3p | miR-23a-3p |  |
| miR-487b-3p | miR-25-5p |  |
| miR-495-5p | miR-26a-5p |  |
| miR-497-5p | miR-299-5p |  |
| miR-502-5p | miR-29a-3p |  |
| miR-512-5p | miR-300 |  |
| miR-513a-3p | miR-301a-5p |  |
| miR-517c-3p+519a-3p | miR-301b-3p |  |
| miR-521 | miR-302a-3p |  |
| miR-522-3p | miR-302d-3p |  |
| miR-543 | miR-30e-5p |  |
| miR-548a-5p | miR-3130-3p |  |
| miR-574-3p | miR-3144-3p |  |
| miR-582-5p | miR-324-3p |  |
| miR-598-3p | miR-346 |  |
| miR-626 | miR-34a-5p |  |
| miR-648 | miR-363-5p |  |
| miR-650 | miR-365a-3p+365b-3p |  |
| miR-6503-5p | miR-376a-3p |  |
| miR-664a-3p | miR-378e |  |
| miR-767-5p | miR-378f |  |
| miR-769-3p | miR-378g |  |
| miR-802 | miR-378h |  |
| miR-887-5p | miR-378i |  |
|  | miR-379-5p |  |
|  | miR-382-3p |  |
|  | miR-411-5p |  |
|  | miR-421 |  |
|  | miR-4286 |  |
|  | miR-4454+7975 |  |
|  | miR-448 |  |
|  | miR-450a-2-3p |  |
|  | miR-4516 |  |
|  | miR-4707-5p |  |
|  | miR-487a-3p |  |
|  | miR-495-3p |  |
|  | miR-496 |  |
|  | miR-498 |  |
|  | miR-499a-5p |  |
|  | miR-514a-3p |  |
|  | miR-514b-5p |  |
|  | miR-519c-3p |  |
|  | miR-520h |  |
|  | miR-542-3p |  |
|  | miR-548ah-5p |  |
|  | miR-548ar-3p |  |
|  | miR-548ar-5p |  |
|  | miR-548e-5p |  |
|  | miR-548g-3p |  |
|  | miR-548v |  |
|  | miR-548y |  |
|  | miR-574-5p |  |
|  | miR-575 |  |
|  | miR-584-3p |  |
|  | miR-585-3p |  |
|  | miR-597-5p |  |
|  | miR-607 |  |
|  | miR-615-3p |  |
|  | miR-627-3p |  |
|  | miR-627-5p |  |
|  | miR-630 |  |
|  | miR-640 |  |
|  | miR-643 |  |
|  | miR-644a |  |
|  | miR-656-3p |  |
|  | miR-663a |  |
|  | miR-764 |  |
|  | miR-765 |  |
|  | miR-888-5p |  |
|  | miR-939-5p |  |

HD: Healthy donor, HNSCC: Head and neck squamous cell carcinoma

**Supplementary Table 2**: Saliva-derived exosomal miRNAs from Volcano plot in Fig. 5B

| **miRNA** | **expression ratio HNSCC / HD** | **p-value** |
| --- | --- | --- |
| **miR-133a-5p** | **0.6605** | **0.0098** |
| **miR-4516** | **0.4460** | **0.0125** |
| **miR-1253** | **0.7601** | **0.0159** |
| **miR-203a-3p** | **0.2540** | **0.0186** |
| **miR-378i** | **0.6872** | **0.0250** |
| **miR-1283** | **0.5616** | **0.0309** |
| **miR-21-5p** | **0.7256** | **0.0309** |
| **miR-378e** | **0.7273** | **0.0461** |
| miR-23a-3p | 0.3253 | 0.0684 |
| miR-16-5p | 0.6359 | 0.0793 |
| miR-29a-3p | 0.7391 | 0.0811 |
| miR-627-5p | 0.8393 | 0.0831 |
| miR-205-5p | 0.3290 | 0.0968 |
| miR-26a-5p | 0.7602 | 0.1101 |
| miR-365a-3p+365b-3p | 0.7079 | 0.1101 |
| miR-200b-3p | 0.6664 | 0.1285 |
| miR-1296-3p | 0.6868 | 0.1422 |
| miR-125b-5p | 0.5932 | 0.1490 |
| miR-1268b | 0.7554 | 0.1490 |
| miR-1246 | 0.6702 | 0.1518 |
| miR-155-5p | 0.5721 | 0.1574 |
| miR-888-5p | 0.8467 | 0.1718 |
| miR-448 | 0.8881 | 0.1827 |
| miR-4286 | 0.8727 | 0.1969 |
| miR-487a-3p | 0.7651 | 0.1969 |
| miR-644a | 0.8729 | 0.1969 |
| miR-498 | 0.8634 | 0.2245 |
| miR-584-3p | 0.7827 | 0.2245 |
| miR-223-3p | 0.2820 | 0.2414 |
| miR-411-5p | 0.7941 | 0.2414 |
| miR-186-5p | 0.8149 | 0.2545 |
| miR-300 | 0.8199 | 0.2545 |
| miR-4707-5p | 0.7913 | 0.2545 |
| miR-597-5p | 0.8154 | 0.2545 |
| miR-148b-3p | 0.8689 | 0.2749 |
| miR-2110 | 0.8044 | 0.2872 |
| miR-30e-5p | 0.8641 | 0.2872 |
| miR-499a-5p | 0.8178 | 0.2872 |
| miR-643 | 0.7799 | 0.2872 |
| miR-630 | 0.7682 | 0.3114 |
| miR-324-3p | 0.7808 | 0.3223 |
| miR-607 | 0.8906 | 0.3223 |
| miR-1272 | 0.8803 | 0.3601 |
| miR-1285-5p | 0.7812 | 0.3601 |
| miR-134-3p | 0.8422 | 0.3601 |
| miR-183-5p | 0.9021 | 0.3601 |
| miR-34a-5p | 0.8620 | 0.3601 |
| miR-363-5p | 0.8671 | 0.3601 |
| miR-585-3p | 0.8775 | 0.3601 |
| miR-615-3p | 0.7257 | 0.3845 |
| miR-4454+7975 | 0.5348 | 0.3929 |
| let-7b-5p | 0.8037 | 0.4003 |
| miR-10b-5p | 0.8858 | 0.4003 |
| miR-1295a | 0.9090 | 0.4003 |
| miR-1323 | 0.8795 | 0.4003 |
| miR-301b-3p | 0.8736 | 0.4003 |
| miR-142-3p | 0.6241 | 0.4378 |
| miR-302d-3p | 0.8107 | 0.4378 |
| let-7f-5p | 1.0800 | 0.4430 |
| miR-1197 | 0.8657 | 0.4430 |
| miR-378g | 0.7845 | 0.4430 |
| miR-2053 | 1.1084 | 0.4880 |
| miR-302a-3p | 0.8887 | 0.4880 |
| miR-542-3p | 0.8962 | 0.4880 |
| miR-1297 | 1.0559 | 0.5353 |
| miR-188-5p | 1.0602 | 0.5353 |
| miR-301a-5p | 0.8935 | 0.5353 |
| miR-450a-2-3p | 0.8609 | 0.5353 |
| miR-548g-3p | 0.8765 | 0.5353 |
| miR-939-5p | 0.8702 | 0.5353 |
| miR-1305 | 0.8352 | 0.5356 |
| miR-575 | 0.8502 | 0.5356 |
| miR-514b-5p | 0.9157 | 0.5846 |
| miR-548e-5p | 0.8835 | 0.5846 |
| miR-548y | 0.8633 | 0.5846 |
| miR-656-3p | 0.8666 | 0.5846 |
| miR-379-5p | 0.9159 | 0.5880 |
| miR-378f | 1.0669 | 0.6331 |
| miR-3130-3p | 1.0149 | 0.6359 |
| miR-378h | 0.9108 | 0.6359 |
| miR-514a-3p | 0.8962 | 0.6359 |
| miR-548ar-3p | 0.8155 | 0.6359 |
| miR-548v | 0.9392 | 0.6359 |
| miR-548ar-5p | 0.9993 | 0.6590 |
| miR-421 | 1.0002 | 0.6888 |
| miR-496 | 1.0507 | 0.6888 |
| miR-1290 | 0.9659 | 0.7433 |
| miR-1972 | 1.0926 | 0.7433 |
| miR-764 | 0.8845 | 0.7433 |
| let-7a-5p | 0.8427 | 0.7990 |
| miR-346 | 1.0101 | 0.7990 |
| miR-520h | 0.9563 | 0.7990 |
| miR-627-3p | 0.9133 | 0.7990 |
| miR-663a | 1.1009 | 0.7990 |
| miR-1255a | 0.9070 | 0.8557 |
| miR-1258 | 0.9825 | 0.8557 |
| miR-376a-3p | 0.9919 | 0.8557 |
| miR-765 | 0.9621 | 0.8557 |
| miR-574-5p | 0.9403 | 0.8773 |
| miR-548ah-5p | 1.0406 | 0.9131 |
| miR-640 | 1.0277 | 0.9131 |
| miR-299-5p | 0.9273 | 0.9385 |
| miR-495-3p | 0.8377 | 0.9385 |
| miR-382-3p | 1.0050 | 0.9710 |
| miR-519c-3p | 0.9254 | 0.9710 |
| miR-199a-3p+199b-3p | 1.0240 | 1.0000 |
| miR-25-5p | 0.9143 | 1.0000 |
| miR-3144-3p | 0.9542 | 1.0000 |

HD: Healthy donor, HNSCC: Head and neck squamous cell carcinoma, **Bold**: significantly differentially present miRNAs between saliva-derived exosomes from HD and HNSCC patients, shown in the waterfall plot in Fig. 5C
